# Different pathologic responses to neoadjuvant anti-PD-1 in primary squamous lung cancer and regional lymph nodes

**DOI:** 10.1038/s41698-020-00135-2

**Published:** 2020-12-01

**Authors:** Yun Ling, Ning Li, Lin Li, Changyuan Guo, Jiacong Wei, Pei Yuan, Fengwei Tan, Xiuli Tao, Shuhang Wang, Zhijie Wang, Ning Wu, Jie Wang, Jianming Ying, Shugeng Gao, Jie He

**Affiliations:** 1 Department of Pathology, National Cancer Center/National Clinical Research Center for Cancer/Cancer Hospital, Chinese Academy of Medical Sciences and Peking Union Medical College, Beijing, China; 2 Clinical Cancer Center, National Cancer Center/National Clinical Research Center for Cancer/Cancer Hospital, Chinese Academy of Medical Sciences and Peking Union Medical College, Beijing, China; 3 Department of Thoracic Surgery, National Cancer Center/National Clinical Research Center for Cancer/Cancer Hospital, Chinese Academy of Medical Sciences and Peking Union Medical College, Beijing, China; 4 Department of PET-CT Center, National Cancer Center/National Clinical Research Center for Cancer/Cancer Hospital, Chinese Academy of Medical Sciences and Peking Union Medical College, Beijing, China; 5 Department of Medical Oncology, National Cancer Center/National Clinical Research Center for Cancer/Cancer Hospital, Chinese Academy of Medical Sciences and Peking Union Medical College, Beijing, China

**Keywords:** Non-small-cell lung cancer, Translational research

## Abstract

Neoadjuvant immunotherapy provides a unique opportunity for understanding therapeutic responses. We analyzed pathologic responses in surgical specimens obtained from 31 squamous non-small cell lung cancer (NSCLC) patients receiving neoadjuvant anti-PD-1 treatment. Fifteen (48.4%) patients achieved pathologic complete response (pCR) or major pathologic response (MPR). Among them, seven (46.7%) were assessed as radiological partial response and eight (53.3%) as stable disease. Among 20 patients with pathologically identified tumor beds in lymph nodes (LNs), 10 and six patients achieved pCR/MPR in primary tumors and paired LNs, respectively. pCR was achieved in 6/19 N1 nodes and 1/7 N2 nodes. Residual viable tumor (RVT) cells in 8/9 MPR specimens had 100% immune-activated phenotype, while a median of 80% of RVT cells in pathologic nonresponse specimens presented immune-excluded/desert phenotype. These findings demonstrated that assessment of pathologic responses in both primary tumor and LNs may be important as a surrogate for assessing neoadjuvant immunotherapeutic efficacy.

## Introduction

Non-small-cell lung cancer (NSCLC) accounts for ~85% of all newly diagnosed lung cancers. The two major histological types of NSCLC are adenocarcinoma and squamous NSCLC, which constitute ~40 and 25–30% of lung cancers, respectively^1–3^. In addition to targeted therapy, recent success with immunotherapies that block the immune inhibition of programmed death 1 (PD-1) protein or its ligand PD-L1 in different tumor types has brought major advances for the treatment of advanced NSCLC^4–7^, including squamous NSCLC. It has been hypothesized that neoadjuvant immunotherapy for early stage NSCLC has the advantage of maximizing T-cell activation using the primary tumor as an antigen source, as well as systemic elimination of micro-metastases, which are the source of postsurgical relapse. Preliminary pilot studies have shown good efficacy with low adverse effects in NSCLC patients with neoadjuvant anti-PD-1 immunotherapies^8,9^, including sintilimab, which is a recombinant humanized anti-PD-1 mAb that blocks interactions between PD-1 and its ligands. This antibody has been tested for safety and activity in patients with advanced solid tumors^10^ and approved in China for lymphoma by the Chinese Center for Drug Evaluation in 2018.

Evaluation of pathologic responses after neoadjuvant chemotherapy has been shown to be a good early surrogate to assess treatment effect and predict survival^11,12^. Unlike the direct killing effects of chemotherapy by cell toxicity, neoadjuvant immunotherapy has a distinct mechanism of indirect tumor eradication by activating tumor-specific T cells, which may lead to different pathologic changes in the anti-PD-1/L1 immune response. Instead of using conventional chemotherapy pathologic response criteria (cPRC), immune-related pathologic response criteria (irPRC) were recently proposed to assess the pathologic features of response to neoadjuvant immunotherapy in resection specimens, showing improved performance compared with cPRC^13^. However, only one study reported on neoadjuvant resected NSCLC with a small cohort size of 20 patients, of which only five were squamous NSCLC. A larger cohort with specific subtypes of NSCLC, such as squamous NSCLC, and patients of different races will make the pathologic assessment criteria more convincing. Additionally, neoadjuvant immunotherapy trials yield abundant tissue samples, including primary tumors and regional lymph nodes collected from surgery after neoadjuvant treatment, which provide a unique opportunity for understanding the responses to immunotherapy and exploring molecular mechanisms of resistance.

In this study, we characterized the pathologic features of neoadjuvant immunotherapy in resected squamous NSCLC according to the irPRC, and reported additional histopathologic findings, especially residual viable tumor (RVT) in lymph nodes and immune-related phenotypes of RVT that may provide further prognostic indication and improve rational therapeutic decisions after surgery.

## Results

### Correlation of RVT with gross pathologic and radiographic tumor bed

**Fig. 1:**
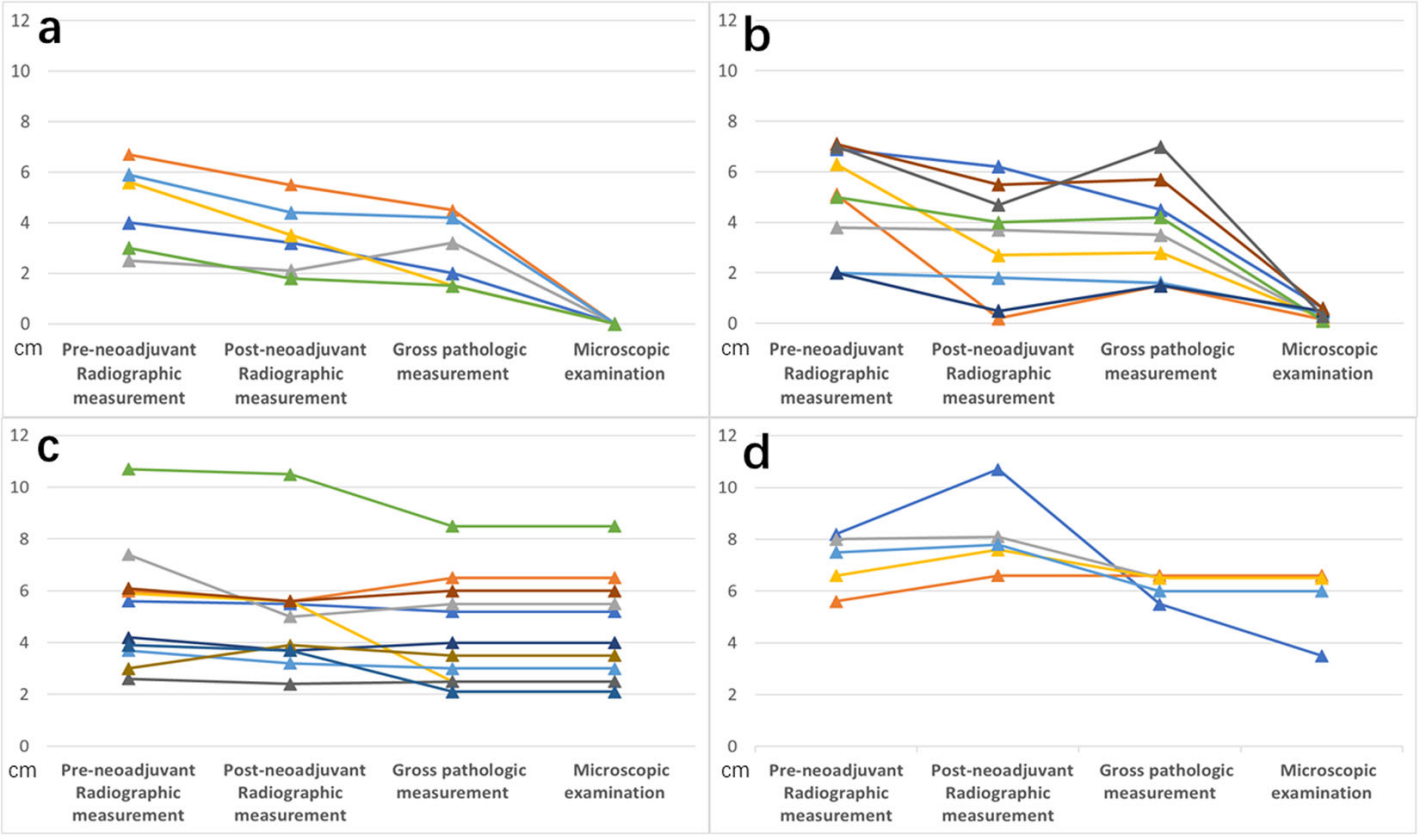
Correlation of microscopic examination with gross pathologic size and radiologic size of the primary tumor after neoadjuvant immunotherapy. **a** patients with pCR. **b** patients with MPR. **c** patients with pPR. **d** patients with pNR. Each line indicates one patient. Although the radiographic tumor size before and after neoadjuvant anti-PD-1 treatment is almost the same as that seen by gross pathologic measurement, it is far from the size of the RVT area observed under the microscope, especially in patients with pCR/MPR. Additionally, only approximately half of the patients with pCR/MPR showed partial response to neoadjuvant immunotherapy in the radiographic evaluation.

**Fig. 2:**
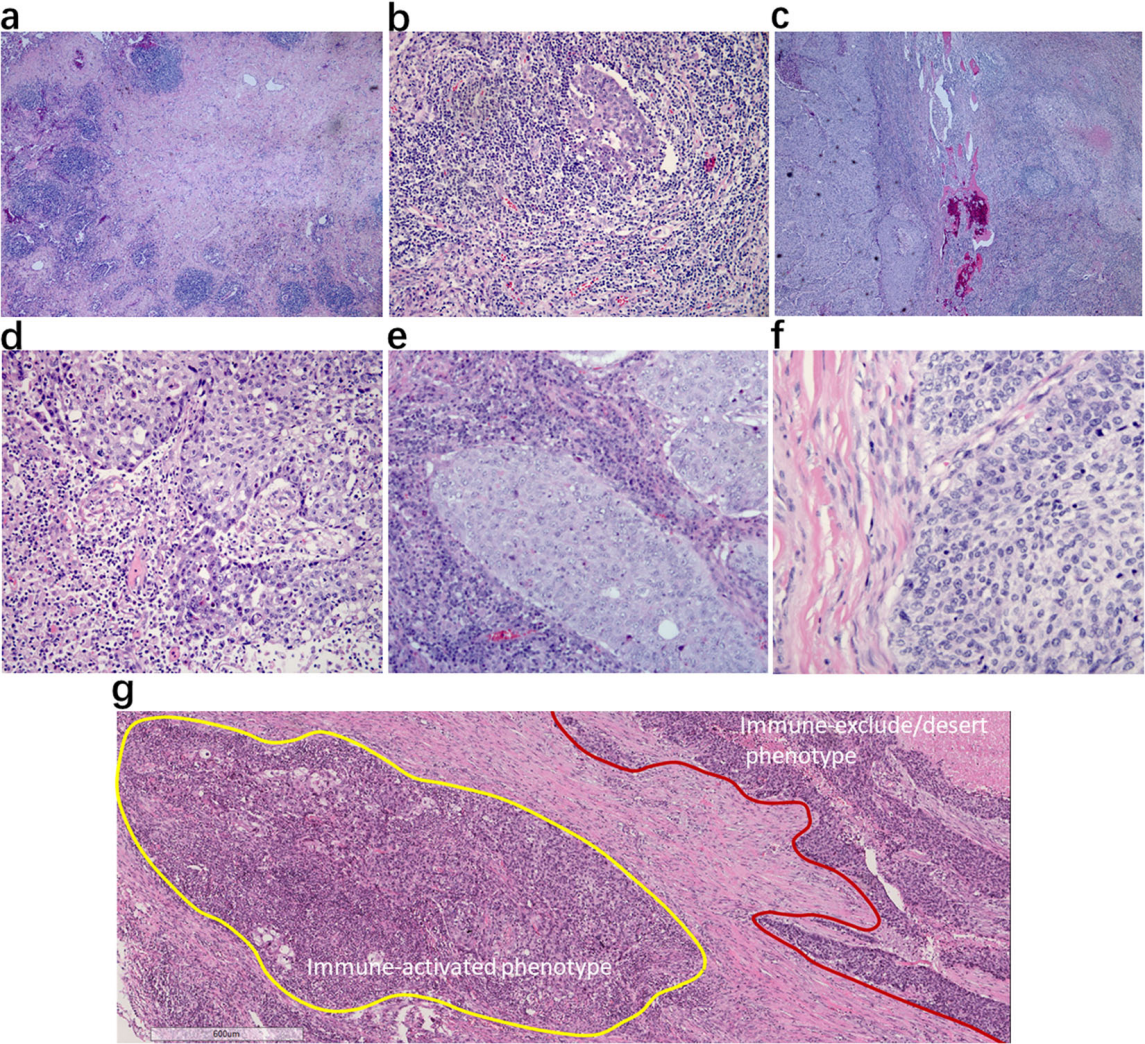
Pathologic responses to neoadjuvant anti-PD-1 in primary tumor specimens of squamous cell NSCLC. **a** Pathologic complete response (pCR). Absence of any viable invasive tumor in the whole tumor bed. **b** Major pathologic response (MPR). Percentage of residual viable tumor (%RVT) is no >10%. **c** Partial pathologic response (pPR). %RVT is >10%. **d**–**f** Tumor-immune histopathologic phenotypes responding (**d**) or non-responding (**e**, **f**) to anti-PD-1 therapy, including **d** immune-activated: presence of different immune cell types both in the parenchyma of tumors and the surrounding stroma, and partial area (lower left, mainly present at the margin of cancer cell nest with an “outside-in” pattern) shows immune-mediated tumor regression; **e** immune-excluded: presence of different immune cell types that cannot penetrate the parenchyma of the tumors but instead are contained in the stroma that surrounds the cancer cells; **f** immune desert: absence of abundant immune cells in either the parenchyma or the stroma of the tumor. **g** Representative heterogeneous responses of different RVT cancer cell nests in the same tumor bed. Original magnifications: (**a**, **c**) ×40, (**b**, **g**) ×100, (**d**–**f**) ×200.

Surgical specimens of post-treatment primary tumor and lymph node were obtained from 31 patients with squamous NSCLC who received neoadjuvant anti-PD-1 therapy. Of the 31 patients, 15 (48.4%) achieved pathologic complete response (pCR)/major pathologic response (MPR) in the primary tumor, with six and nine achieving pCR (19.4%) and MPR (29.0%), respectively; 11 (35.5%) had a partial pathologic response (pPR) and five (16.1%) had no pathologic response (pNR; Figs. 1–2 and Supplementary Table 1). Among the 15 patients with pCR/MPR, seven (46.7%) were assessed as radiological partial response and eight (53.3%) had stable disease. The median pre-neoadjuvant radiographic tumor size in patients who achieved pCR/MPR was smaller (median, 5.1 cm; range, 2.0–7.1 cm) than that of pPR (median, 6.0 cm; range, 2.6–10.7 cm), or pNR (median, 7.5 cm; range, 5.6–8.2 cm; Fig. 1), suggested that patients with smaller pre-treatment radiographic tumor size tend to have better pathologic responses.

### Features of immune-related regression in the resected specimens according to irPRC

Histologic features of irPRC in the regression bed mainly include lymphoid infiltrates/aggregates, tertiary lymphoid structures (TLSs), dense plasma cells, interstitial foamy macrophages, cholesterol clefts, proliferative fibrosis, and neovascularization. All these features were observed in the resected primary tumors in our cohort, with lymphoid infiltrates/aggregates, TLSs, dense plasma cells, and neovascularization being the most commonly seen features (Supplementary Table 1 and Supplementary Fig. 1), followed by granulomas, giant cells, necrosis, and alveolar foamy macrophages. Giant cells were mainly related to keratin, while alveolar foamy macrophages were mainly related to obstructive pneumonia. Necrosis was not common (1/6) in specimens with pCR. The distinct features of immune-mediated regression in the primary tumor were also observed in the resected tumor-draining lymph nodes, even in those without RVT, where a regression bed was identified (Supplementary Fig. 2).

### Heterogeneous responses of RVT

**Fig. 3:**
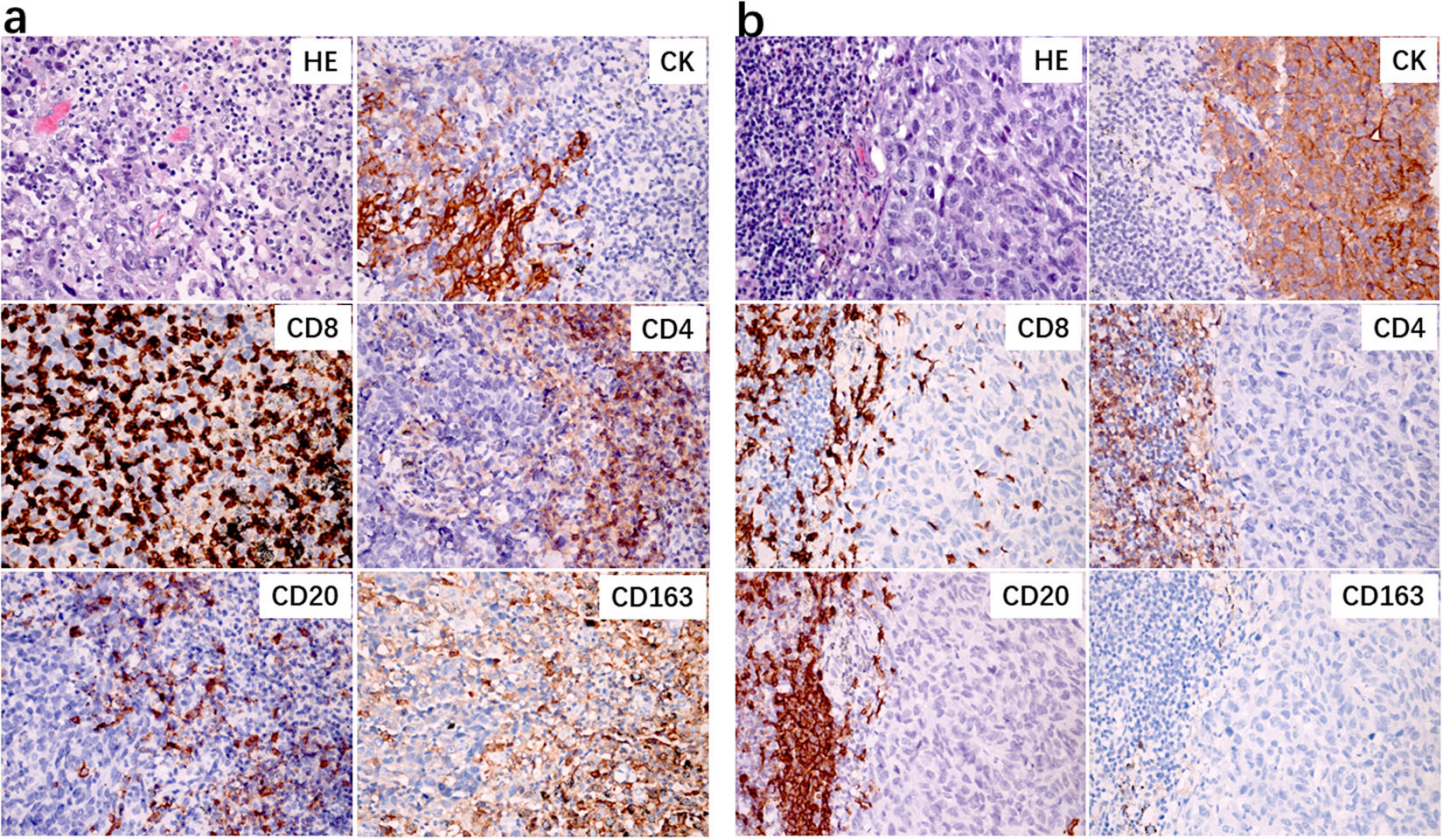
Heterogeneous responses of RVT in different regional lymph nodes. **a** Histological morphology and immune cell distribution in an inflamed morphological phenotype of RVT in one resected N1 lymph node, showing infiltration with many immune cells (H&E), high intensity of CD8 + T cells, infiltration of CD4 + T cells, CD20 + B cells, and CD163 + histocytes in CK(AE1/AE3) + RVT cell nests. **b** Histological morphology and immune cell distribution in a non-inflamed morphological phenotype of RVT in one resected N2 lymph node from the same patient, showing infiltration of few immune cells (H&E), low intensity of CD8 + T cells, no infiltration of CD4 + T cells, CD20 + B cells, and CD163 + histocytes in CK + RVT cell nests. Original magnification: ×200.

Differing extents of immune-mediated tumor response among cancer cell nests was found in the same tumor. As for RVT, we identified that there were three histopathological immune phenotypes of immune-related morphological changes in response to anti-PD-1 immunotherapy: immune-activated, immune-excluded, and immune-desert (Fig. 2d–f). Heterogeneous immune responses were identified among different RVT tumor cell nests in the same tumor (Fig. 2g). In parallel with histological morphology, immunohistochemistry staining results showed that different intensities of infiltrating CD8 + T cells were present in immune-activated RVTs. As shown in Supplementary Table 1, RVT in 8/9 MPR specimens showed 100% immune-activated phenotype, and the remaining MPR specimen demonstrated 80% immune-activated phenotype. In contrast, 60% immune-excluded/desert phenotype (range, 0–90%) was observed in specimens with pPR, and 80% immune-excluded/desert phenotype (range, 10–90%) was found in pNR. One specimen with pPR had a 100% immune-activated phenotype. Additionally, as for immune-activated RVT, we also identified two histological morphological phenotypes and immunophenotypes (inflamed and non-inflamed). As shown in Fig. 3, the inflamed phenotype presented with infiltration of many immune cells and increased intensity of CD8+T cells, infiltration of CD4+T cells, B cells (CD20+), and histocytes (CD163+) in the cancer cell nest, determined by immunohistochemistry (Fig. 3a), when compared with non-inflamed immune-activated RVT (Fig. 3b). No significant difference was found in PD-L1 expression on RVT tumor cells; however, increased intensity of PD-L1-expressing immune cells was identified in RVT with inflamed phenotype (Supplementary Fig. 3).

### Heterogeneous responses of RVT in resected lymph node specimens

**Fig. 4:**
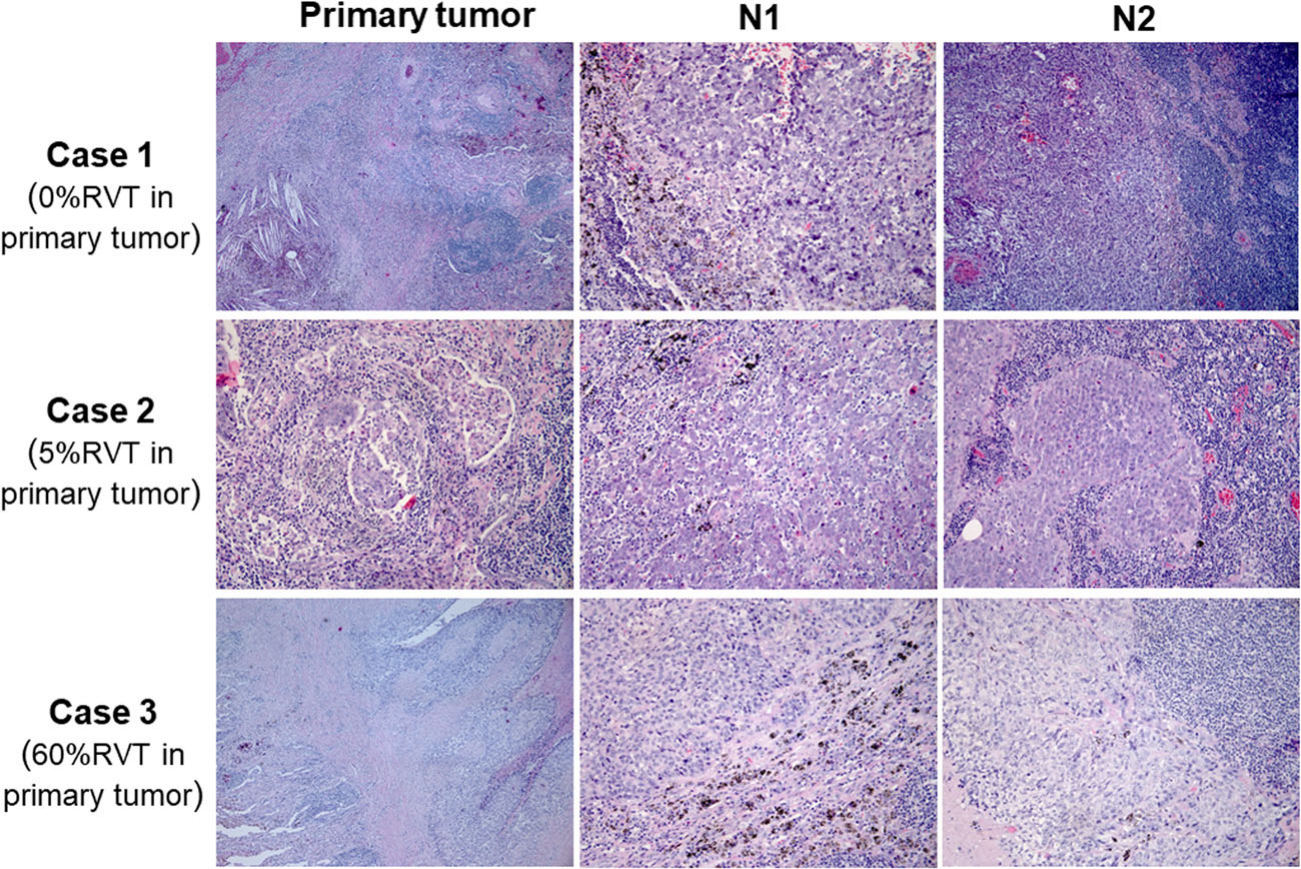
Different pathologic responses of RVT in primary and paired regional lymph nodes. Histologic phenotypes of primary tumors and paired regional lymph nodes from three patients. Case 1 showed no RVT in the primary tumor, with inflamed immune-activated phenotype in the N1 lymph node, but non-inflamed phenotype in the N2 lymph node. Case 2 showed 5% irRVT in the primary tumor, with inflamed immune-activated phenotype in the N1 lymph node, but non-inflamed phenotype in the N2 lymph node. Case 3 showed 60% irRVT in the primary tumor, with non-inflamed phenotype both in N1 and N2 lymph nodes.

Resected lymph nodes from 20 patients were pathologically identified to have tumor beds of pre-treatment metastatic cancer (Supplementary Table 1). Thirteen patients had tumor beds in the N1 node only, one patient in N2 node only, and six patients in both N1 and N2 nodes. Immune-related RVT in lymph nodes (irRVT-LN) was identified in a range of 0–100% in these 20 patients. Among the 20 patients with pre-treatment lymph node metastasis, six had 0–10% irRVT-LN (six pCR-LN and 0 MPR-LN) in lymph nodes, while 10 had 0–10% irRVT (four pCR and six MPR) in paired primary tumors. In total, 6 out of 19 patients with metastasis in N1 lymph nodes achieved pCR-LN, while only 1 out of 7 patients with metastasis in N2 lymph nodes achieved pCR-LN. Both phenotypes of immune-activated and excluded/desert were identified in the irRVT-LN (Supplementary Fig. 4 and Supplementary Table 1). Additionally, in four patients with RVT identified both in N1 and N2 lymph nodes, the percentage of inflamed morphological phenotype in the N1 lymph node was higher than the N2 lymph node for each patient (Figs. 3 and 4).

## Discussion

Pathologic response evaluation after neoadjuvant therapy provides a unique window at an early timepoint to evaluate curative efficacy, and potentially predict disease-free and overall survival^14,15^. Compared with the well-recognized pathologic response evaluation for neoadjuvant chemotherapy, the preliminarily irPRC has been recently defined by Cottrell and colleagues based on a pilot trial of neoadjuvant anti-PD-1 treatment in a small cohort of 20 NSCLC patients^13^, in which 45% of patients achieved pCR/MPR, particularly in adenocarcinoma, with 50% (6/12) pCR/MPR, but only 33.3% (2/6) for squamous NSCLC. Therefore, the efficacy of neoadjuvant immunotherapy for NSCLC needs to be further assessed in clinical trials and the corresponding irPRC require verification with larger numbers of cases, while taking into consideration the tumor subtype and patient’s race.

Recently, Gao et al.^8^ reported another clinical trial of neoadjuvant anti-PD-1 treatment for NSCLC, in which 38 Chinese patients were enrolled, mainly with squamous NSCLC (31 cases). The overall pCR/MPR rate was similar to the trial reported by Forde and colleagues (45% vs 40.5%)^9^, however, different pCR/MPR rates were observed in the histological subtypes. The pCR/MPR rate for squamous NSCLC was 48.4% (15/31) and 0% (0/6) for adenocarcinoma in the Chinese NSCLC cohort, which differed from Western patients (33.3%, 2/6, and 50%, 6/12, respectively). Further trials with larger cohort are needed to confirm this preliminary finding. Mechanisms underlined is also interested to be further explored.

We found that using traditional imaging evaluation after neoadjuvant immunotherapy, to assess therapeutic effect on tumor cells, can be challenging and misleading because it does not necessarily reflect the actual therapeutic effect. This has been reported in many immunotherapy studies, where patients with pseudo progression of imaging were even seen. In our cohort, only seven of the 15 patients who achieved pCR/MPR showed partial response in imaging. Tao and colleagues reported that there was a significant correlation between the pathological response and the 18F-FDG PET-CT responses that were classified using PERCIST^15^. We also found that for primary tumors, the tumor size in patients with pCR/MPR was smaller than that of pNR, which was consistent with the recent retrospective analyses showing that in the advanced metastatic treatment setting, patients with lower tumor burdens were more likely to experience long-term survival after anti-PD-1 therapy^16^. Forde and colleagues also showed that patients with earlier stage (I–II) NSCLC achieved higher pCR/MPR rates compared with patients with stage IIIA (7/14 vs 2/7) after two doses of neoadjuvant anti-PD-1 immunotherapy^9^.

In our cohort, the histologic features of pathological response after neoadjuvant immunotherapy were consistent with those reported by Cottrell and colleagues^13^. In particular, immunotherapy-related necrosis mainly manifested as sudden death of the whole cell nest, caused by the destruction of stroma that provides nutrition for tumor cells. Tumor cell fragments were quickly phagocytized by macrophages to form granulomas. Therefore, necrosis is not common in specimens with pCR. However, most necrosis of squamous NSCLC is not caused by immunotherapy. Necrosis is common in untreated squamous NSCLC, especially in large tumors, therefore it is difficult to distinguish between necrosis caused by the treatment and pre-existing necrosis, ultimately affecting the %RVT value. It suggests that, although establishment of a standard irPRC for pan-cancer purpose was proposed^17^, it should also reflect the characteristics of different tumor types. Some histological features from special cases were noted in our study. Among the six pCR specimens, residual squamous cell in situ carcinomas were identified in two specimens. Residual squamous cell in situ carcinoma was not classified as RVT. One pre-treatment radiographically assessed cT1aN0 patient who was confirmed as a pT1aN0 by pathologic examination experienced pPR. Histopathologic examination demonstrated this tumor mainly presented an endotracheal growth pattern. This growth pattern may not respond quickly to immunotherapy because only part of the tumor surface is accessible to immune cells.

The presence of a histopathological growth pattern indicates cancer cell nests surrounded by stroma. We noticed differing extents of immune-mediated tumor responses among cancer cell nests in the same tumor bed, which may be due to inter-tumor heterogeneity, genetics, or tumor microenvironment. Three histological immune phenotypes were classified: immune-activated, immune-excluded, and immune desert. Different cancer cell subclone responses to PD-1 inhibitors are different and the time required for tumor cell clearance also varies. Therefore, at the same timepoint, different tumor cells will present different histopathologic morphologies. Some tumor cells will have been cleared by the immune response, leaving the regression bed, while some may only have been partially cleared with still others becoming activated (including the existing immune response and reactivation after treatment, with the latter being predominant), while some show immune rejection or no response. Accordingly, in some patients with pPR and pNR, their RVT cells showed large numbers of infiltrating immune cells (inflamed), and began to gradually clear tumor cells in an “outside-in” way, in which regression beds were observed surrounding residual tumor (Fig. 2d, g)^13^, indicating that these tumor cells are immunogenic and effective for neoadjuvant immunotherapy drugs. However, the drug takes effect later and clearance of cancer cells need more time, compared with chemotherapy. That means a specific interval between the initiation of neoadjuvant immunotherapy and surgery is critical for enhanced systemic anti-tumor effect. It may be different among individuals or tumor types. Performing surgery too soon after immunotherapy initiation or waiting too long diminished the neoadjuvant effect. Therefore, using only %RVT for pathological evaluation of immunotherapy patients is not sufficient, and the histologic phenotype of the RVT immune response should also be evaluated. The identification of the histopathological immune phenotypes of RVT may be of great significance to guide the choice of follow-up immunotherapy, chemotherapy or radiotherapy.

**Fig. 5:**
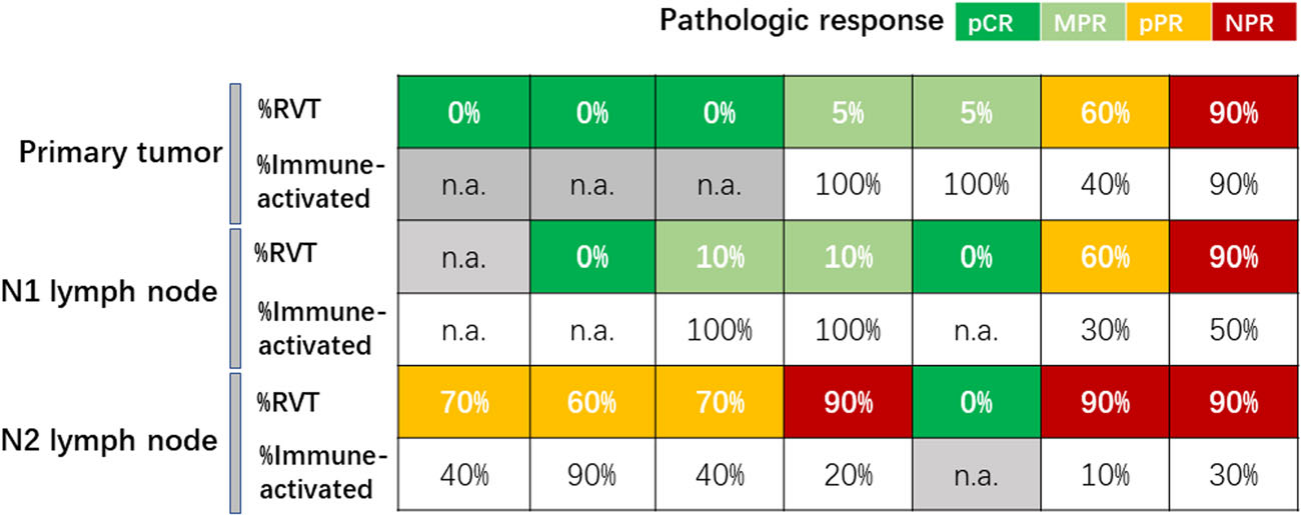
Different pathologic responses in primary tumor and lymph nodes. Different pathologic responses of RVT to anti-PD-1 treatment in primary and regional lymph nodes of patients with both pathologically identified pre-treatment N1 and N2 lymph node metastasis.

Another important issue is the evaluation of the pathological response of lymph node metastasis. In our group, three of six primary pCR patients had pathologic lymph node metastasis, especially in the N2 lymph nodes, demonstrating that evaluation of pathological response of lymph nodes is also very important (Fig. 5). These results suggest that additional studies should be designed to demonstrate whether a long interval between pre-surgery treatment and surgery or a period of continued immunotherapy after surgery may further boost anti-tumor immune responses and avert tumor relapse, especially in N2 lymph nodes, while additional therapy (chemotherapy and/or radiotherapy) might be needed for patients harboring RVT with immune-excluded/desert phenotype.

Taken together, the neoadjuvant trial affords a unique opportunity for understanding the responses to immune checkpoint blockade therapies and carrying out reverse translation, given that there is a rapid and efficient method of gaining high-quality specimens to examine primary resistance to immunotherapies and identify signatures of responders^18^. Standardized irPRC based on distinctive irPR features is needed to assess neoadjuvant immunotherapies in on-going clinical trials. We suggest that irPRC of both the primary tumors and lymph nodes should be assessed, as well as the immune-related histological phenotype of the RVT cells, which may carry prognostic significance but needs further study for correlation with clinical outcome. The use of irPRC and immune-related phenotypes of RVT as a surrogate for long-term patient outcomes require further validation in additional studies with larger cohorts and long-term follow-up.

## Methods

### Patient selection and data collection

Surgical specimens of post-treatment primary tumor and lymph node were obtained from 31 patients with squamous cell NSCLC who were recruited in a phase Ib study of neoadjuvant anti-PD-1 (sintilimab) therapy at the National Cancer Center/Cancer Hospital, Chinese Academy of Medical Sciences, Beijing, China (Registration Number: ChiCTR-OIC-17013726)^8^. Patients received two doses of intravenous sintilimab (200 mg) every 3 weeks (Q3W), followed by surgery within 29–43 days after the first dose of sintilimab. The study was approved by the Ethics Committee and Institutional Review Boards of Cancer Hospital, Chinese Academy of Medical Sciences and Peking Union Medical College, and all patients signed informed consent. Data regarding imaging, clinical characteristics and treatment history were extracted from the medical records system.

### Gross pathologic examination and histologic assessments

Briefly, the tumor bed was identified and measured. The tumor bed was sampled I its entirety if it was small (≤3 cm). In cases where the tumor bed was >3 cm, a minimum of one section per diameter of the tumor bed was sampled. However, if ≤10% RVT was seen in the initial histologic sampling, all remaining bed was sampled. Hematoxylin and eosin (H&E)-stained slides from both primary tumor and lymph node were assessed histologically according to the immune-related pathologic response criteria (irPRC) developed by Cottrell et al.^13^ irPRC incorporates the regression bed as a major feature, which is specifically defined by proliferative fibrosis with neovascularization and evidence of immune activation and cell death. In this system, the tumor bed is defined as the regression bed + RVT + necrosis.

### Immunohistochemistry

Serial 5 mm tissue sections from resected primary tumors and lymph nodes were deparaffinized, rehydrated, pretreated for antigen retrieval, and stained with Ready-to-use (RTU) primary antibodies (CD20[Clone L26, Ready-to-use, Kit-0001, Maxim Biotechnologies, Fuzhou, China], CD3[Clone SP7, Ready-to-use, Kit-0003, Maxim Biotechnologies, Fuzhou, China], CD4[Clone UMAB64, Ready-to-use, ZM-0418, Zhongshan Golden Bridge Biotechnologies, Beijing, China], CD8[Clone SP16, Ready-to-use, ZA-0508, Zhongshan Golden Bridge Biotechnologies, Beijing, China], CD163[Clone 10D6, Ready-to-use, ZM-0428, Zhongshan Golden Bridge Biotechnologies, Beijing, China], CK[Clone AE1/AE3, Ready-to-use, Kit-0009, Maxim Biotechnologies, Fuzhou, China], and PD-L1[Clone 22C3 pharmDx, Ready-to-use, SK006, DAKO]) on a DAKO Link48 Autostainer (DAKO) with respective detection kits.

### Calculation of percentage of immune-related residual viable tumor (%irRVT) score

Percentage of irRVT was determined by summing tumor area and tumor bed area across all slides^13^:

1 $$\% {\mathrm{irRVT}} = {\mathrm{sum}}\,{\mathrm{of}}\,{\mathrm{viable}}\,{\mathrm{tumor}}\,{\mathrm{area}}/{\mathrm{total}}\,{\mathrm{tumor}}\,{\mathrm{bed}}\,{\mathrm{area}}\,({\mathrm{regression}}\,{\mathrm{bed}} + {\mathrm{RVT}} + {\mathrm{necrosis}}) \times 100\%$$

The three components of the tumor bed were evaluated separately. According to the %irRVT value of the primary tumor, patients were grouped as having a pCR (pathologic complete response, absence of any viable invasive tumor), MPR (major pathologic response, irRVT≤10%), pPR (partial pathologic response, 10%<%irRVT<90%), or pNR (no pathologic response, irRVT≥90%). The same calculation was applied to lymph nodes (Supplementary Fig. 5). However, if more than one lymph node was found to have pre-treatment tumor deposit (RVT and/or regression bed), the final %irRVT-LN equaled the sum of %irRVT of each affected lymph node divided by the total number of affected lymph nodes, provided they were located in different tumor beds.

### Identification of histopathological immune phenotypes of RVT

Owing to the heterogeneity of tumor cells/nests/subclones, where the gene expression profile and immune microenvironment are different, the immune responses to anti-PD-1 inhibitors may differ and the time required to generate a response might also vary. Therefore, at the same timepoint, different tumor cells/nests of RVT in the same tumor bed show different histopathological morphology, which we termed “histopathological immune phenotype”. By referring to the tumor-immune phenotypes of biopsy specimens before treatment^19,20^, and based on the density of tumor infiltrating immune cells within a tumor nest and its surrounding stroma, three histopathological immune phenotypes were classified: immune-activated, immune-excluded, and immune desert. The immune-activated phenotype is characterized by the presence of immune cells both in the parenchyma of the tumor nest and the surrounding stroma, accompanied by different levels of irregular tumor-stroma interface, infiltrated by dense immune cells or replaced by regression tissue, or both. The immune-activated phenotype indicates that the tumor cells are antigenic and have been activated by the immune response following PD-1 blockade treatment. However, additional time or immunotherapy is needed to complete tumor clearance. The immune-excluded phenotype is characterized by the presence of different immune cell types that cannot penetrate the parenchyma of the tumor nests but instead are contained in the stroma that immediately surrounds the cancer cells. The immune-desert phenotype is characterized by the absence of abundant immune cells in either the parenchyma or the adjacent peritumoral stroma of tumor nests. Each phenotype was identified and their respective percentages of RVT were evaluated, both in primary tumor and lymph nodes.

### Reporting summary

Further information on research design is available in the Nature Research Reporting Summary linked to this paper.

## Supplementary information

Supplementary Information

Reporting Summary Checklist

## Data Availability

The data generated and analysed during this study are described in the following data record: 10.6084/m9.figshare.13072436^21^. The histology images (in jpg file format) supporting the findings of this study, are publicly available in the figshare repository, as part of the above data record. Neoadjuvant anti-PD-1 patient clinical data and the slide images of each post-treatment resected specimen, used in this study, are available on reasonable request from the corresponding author, Dr. Jianming Ying, email address: jmying@cicams.ac.cn.

## Code availability

The code that support the findings of this study are available from the corresponding author on reasonable request.
