# Supplementary material for: Different pathologic responses to neoadjuvant anti-PD-1 in primary squamous lung cancer and regional lymph nodes
Source: NPJ Precis Oncol. 2020 Dec 1;4:32. doi: 10.1038/s41698-020-00135-2 (PMC7708412; doi:10.1038/s41698-020-00135-2)
Supplement: Supplementary file 1 — Supplementary Information [file 41698_2020_135_MOESM1_ESM.pdf]

Supplementary Figures and Table

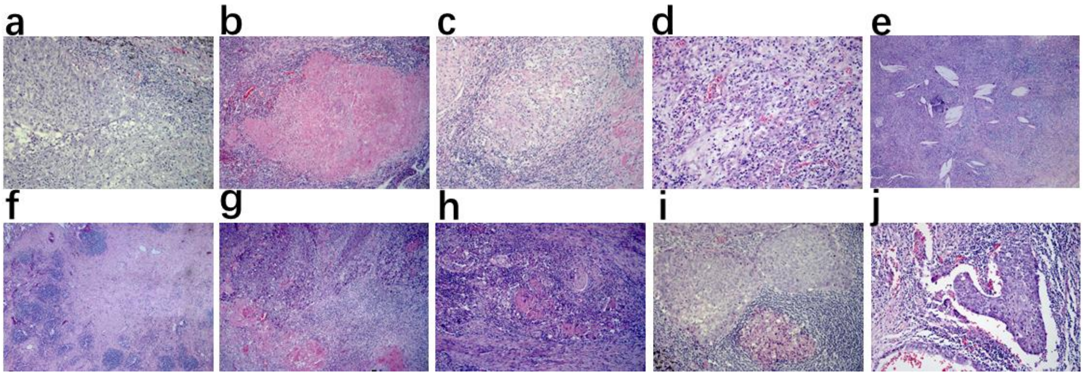

Supplementary Fig. 1 Histologic features of pathologic response to neoadjuvant anti-PD-1 in primary tumor specimens of squamous cell NSCLC. a–f Immune-related pathological features in the regression bed. **a** Partially immune-responding cancer cell nest (lower right region involved by dense lymphocytes, macrophages and proliferative fibrosis supporting the immune-mediated tumor regression). **b** Massive tumor cell death. **c** Granuloma. **d** Neovascularization and proliferative fibrosis. **e.** Cholesterol clefts. **f.** Lymphoid aggregates and tertiary lymphoid structures (TLSs). **g–j** Immune-related pathological features of residual viable tumor. **g** A partially responding keratinizing squamous cell cancer. **h** A regression bed containing dense plasma cells and giant cells caused by residual keratin pearls. **i** Heterogeneous immune responses of different cancer cell nests in the same tumor. **j** Residual viable tumor cells in an MPR specimen. Original magnifications: (a–d, g–j) ×100, (e, f) ×40.

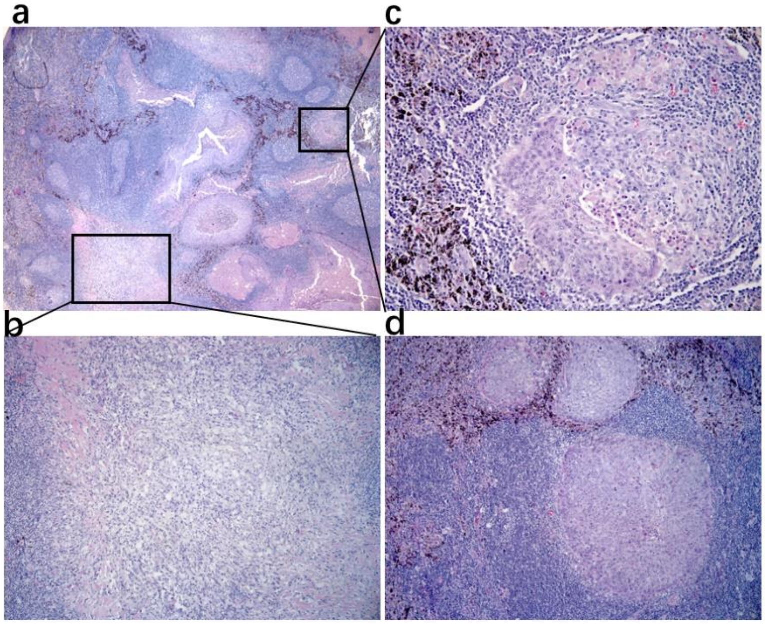

Supplementary Fig. 2 Heterogeneous responses of RVT to anti-PD-1 treatment in lymph node. a Heterogeneous pathologic responses of different residual viable tumor cell nests in resected lymph node. **b** Regression bed. **c** Partial immune-mediated pathologic responses in one RVT cell nest. **d** RVT cells with no pathologic response. Original magnifications: (a) ×40, (b–d) ×100.

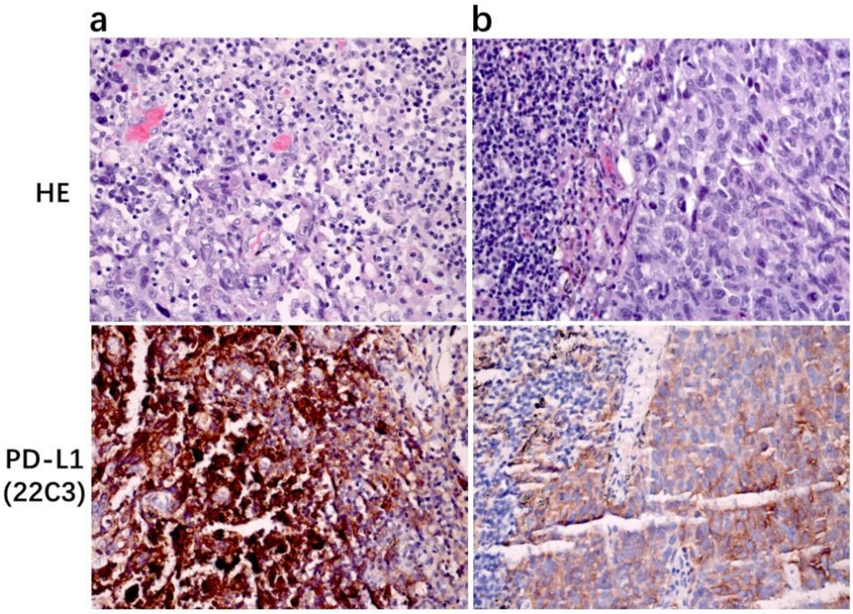

Supplementary Fig. 3 PD-L1 expression on RVT tumor cells and immune cells in different lymph nodes. a Inflamed morphological phenotype of RVT in one resected N1 lymph node showed high intensity of PD-L1-expressing immune cells in RVT cell nests. **b** Non-inflamed morphological phenotype of RVT in one resected N2 lymph node from the same patient showed no significant difference in PD-L1 expression on RVT tumor cells. Original magnifications: ×200.

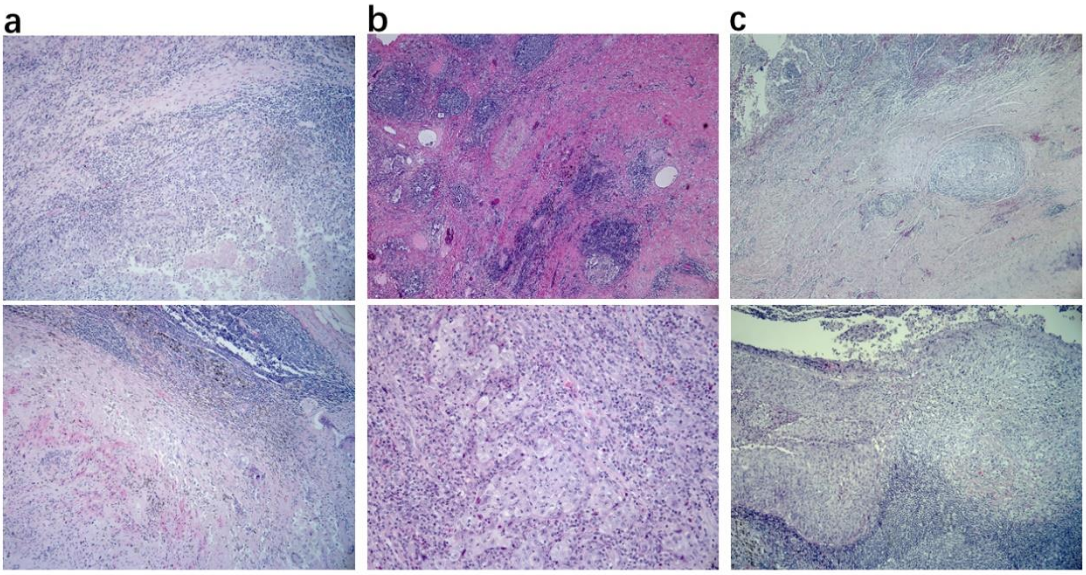

Supplementary Fig. 4 Features of immune-mediated pathologic response in paired primary tumors and lymph nodes from pCR patients. Three of six primary pCR patients were found to have RVT in their dissected draining lymph nodes. **a** Similar histologic features of the regression bed were found both in the primary tumor (top panel) and paired lymph node (bottom panel) as shown. No residual viable tumor cell was identified. **b-c** Two primary pCR patients with residual viable tumor cells in the dissected lymph nodes are shown. Presence of partial immune-mediated pathologic responses in the lymph node metastatic cancer cells (bottom panel) from both patients. The histologic features indicate the tumor cells are immune-activated in **b** (center of the bottom panel), but immune-excluded in the RVT and regression bed in **c** (left and right regions of the bottom panel, respectively). Original magnifications: (a) ×100 (top panel), ×200 (bottom panel), (b) ×100 (top panel), ×200 (bottom panel), (c) ×100 (top panel), ×200 (bottom panel).

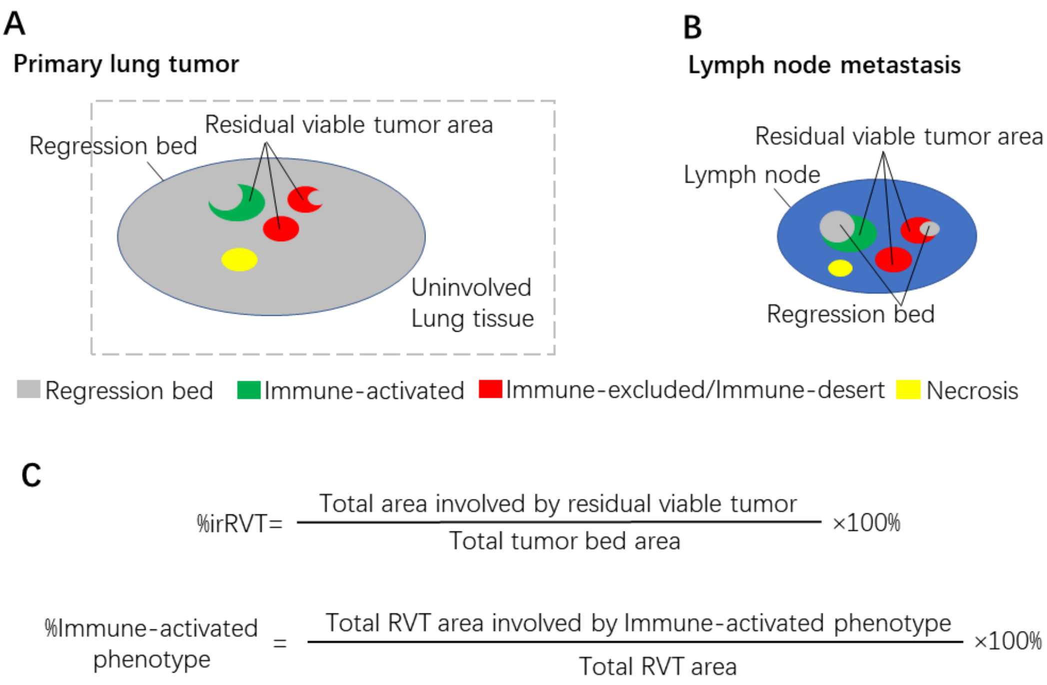

Supplementary Fig. 5 Proposal for quantitative irRVT and immune histopathologic phenotypes of RVT. The regression bed area, RVT area, and areas of necrosis are identified both in primary lung tumor (**A**) and lymph nodes (**B**). Also the immune histopathological phenotypes of RVT includes immune-activated, immune-excluded/immune-desert, which reflect the immune response state of RVT cells to anti-PD-1 at the time of surgery. **C** %irRVT is assessed by dividing the total surface area of RVT by the total tumor bed area ×100. The total tumor bed area is composed of the regression bed area + RVT area +areas of necrosis. If multiple foci of residual tumor are present, these are added together for the RVT area, provided they are located in a single tumor bed. The lymph node %irRVT (irRVT-LN) is calculated separately for each lymph node affected, but using the same approach; that is, by measuring the total area of RVT and dividing by total tumor bed area for each lymph node. However, if more than one lymph node is identified to have pre-treatment tumor deposit (RVT and/or regression bed), the final %irRVT-LN=sum of %irRVT of each affected lymph nodes divided by the total number of affected lymph nodes, provided they are located

in different tumor beds.

Supplementary Table 1

Patient No.	3	14	18	25	31	33	8	11	12	16	23	24	32	34	39	1	2	5	9	13	20	22	27	28	30	40	4	10	21	37	38
**Pathologic response of primary tumor**	pCR						MPR									pPR											pNR				
**Features in the regression bed**																															
Tertiary lymphoid structures	3+	3+	3+	3+	3+	3+	3+	3+	3+	3+	3+	3+	3+	3+	3+	2+	2+	3+	2+	1+	2+	2+	3+	1+	1+	3+	2+	1+	2+	1+	1+
Lymphoid aggregates	+	+	+	+	+	+	+	+	+	+	+	+	+	+	+	+	+	+	+	+	+	+	+	+	+	+	+	+	+	+	+
Proliferative fibrosis	+	+	+	+	+	+	+	+	+	+	+	+	+	+	+	+	+	+	+	+	+	+	+	+	+	+	+	+	+	+	+
Neovascularization	+	+	+	+	+	+	+	+	+	+	+	+	+	+	+	+	+	+	+	+	+	+	+	+	+	+	+	+	+	+	+
Dense plasma cells	+	+	+	+	+	+	+	+	+	+	+	+	+	+	+	+	+	+	+	+	+	+	+	+	+	+	+	+	+	+	+
Interstitial foamy macrophages	-	+	-	-	-	+	+	+	+	+	-	+	+	+	+	+	+	+	-	-	+	-	+	-	+	-	+	-	-	-	-
Cholesterol clefts	-	+	-	-	+	-	+	-	+	+	-	+	-	-	+	+	+	+	-	+	-	-	+	-	-	+	+	-	-	+	-
**Composition of primary tumor bed**																															
RVT %	0	0	0	0	0	0	5	5	10	5	5	5	5	10	5	70	80	40	60	60	80	50	40	70	60	50	90	90	90	90	90
Necrosis %	0	40	0	0	0	0	50	10	40	10	10	5	0	60	35	10	10	20	20	10	10	10	10	10	20	10	5	5	5	5	5
Tumor stroma/regression bed %	100	60	100	100	100	100	45	85	50	85	85	90	95	30	60	20	10	40	20	30	10	40	50	20	20	40	5	5	5	5	5
**Immune phenotypes of RVT**																															
Immune excluded/desert %	na	na	na	na	na	na	20	0	0	0	0	0	0	0	0	60	20	0	60	60	60	30	10	20	60	90	10	80	10	90	90
Immune activated %	na	na	na	na	na	na	80	100	100	100	100	100	100	100	100	40	80	100	40	40	40	70	90	80	40	10	90	20	90	10	10
**Regional LN information**																															
**Pathologic response of regional LN**	na	pCR	pPR	pPR	pPR	na	pCR	pPR	pPR	pCR	pCR	na	na	na	pCR	na	na	na	pPR	pPR	pNR	pPR	pPR	na	na	pCR	pNR	na	pNR	pNR	pPR
LN with regression bed/RVT	N0	N1	N2	N1+N2	N1+N2	N0	N1	N1+N2	N1	N1	N1	N0	N0	N0	N1+N2	N0	N0	N0	N1+N2	N1	N1	N1	N1	N0	N0	N1	N1	N0	N1+N2	N1	N1
LN with RVT (ypN)	N0	N0	N2	N1+N2	N1+N2	N0	N0	N1+N2	N1	N0	N0	N0	N0	N0	N0	N0	N0	N0	N1+N2	N1	N1	N1	N1	N0	N0	N0	N1	N0	N1+N2	N1	N1
RVT %	na	0	70	40	50	na	0	70	80	0	0	na	na	na	0	na	na	na	60	60	90	70	40	na	na	0	90	na	90	100	60
Immune excluded % in RVT	na	0	60	10	40	na	0	40	90	0	0	na	na	na	0	na	na	na	60	60	95	80	5	na	na	0	90	na	10	90	95
Immune activated % in RVT	na	0	40	90	60	na	0	60	10	0	0	na	na	na	0	na	na	na	40	40	5	20	95	na	na	0	10	na	90	10	5
**Primary tumor size**																															
Pre-neoadjuvant Radiographic measurement(cm)	4	6.7	2.5	5.6	5.9	3	6.9	5.1	3.8	6.3	2	5	2	7.1	7	5.6	6	7.4	5.9	3.7	10.7	4.2	6.1	2.6	3	3.9	8.2	5.6	8	6.6	7.5
Post-neoadjuvant Radiographic measurement(cm)	3.2	5.5	2.1	3.5	4.4	1.8	6.2	0.2	3.7	2.7	1.8	4	0.5	5.5	4.7	5.5	5.6	5	5.6	3.2	10.5	3.7	5.6	2.4	3.9	3.7	10.7	6.6	8.1	7.6	7.8
Gross pathologic measurement(cm)	2	4.5	3.2	1.5	4.2	1.5	4.5	1.5	3.5	2.8	1.6	4.2	1.5	5.7	7	5.2	6.5	5.5	2.5	3	8.5	4	6	2.5	3.5	2.1	5.5	6.6	6.5	6.5	6
Microscopic examination(cm)	0	0	0	0	0	0	0.6	0.15	0.15	0.2	0.4	0.1	0.5	0.6	0.3	5.2	6.5	5.5	2.5	3	8.5	4	6	2.5	3.5	2.1	3.5	6.6	6.5	6.5	6
RECIST	SD	SD	SD	PR	SD	PR	SD	PR	SD	PR	SD	SD	PR	PR	PR	SD	SD	PR	SD	SD	SD	SD	SD	SD	PD	SD	PD	SD	SD	SD	SD
Pathologic response	pCR	pCR	pCR	pCR	pCR	pCR	MPR	MPR	MPR	MPR	MPR	MPR	MPR	MPR	MPR	pPR	pPR	pPR	pPR	pPR	pPR	pPR	pPR	pPR	pPR	pPR	pNR	pNR	pNR	pNR	pNR
