# Supplementary material for: Different pathologic responses to neoadjuvant anti-PD-1 in primary squamous lung cancer and regional lymph nodes
Source: NPJ Precis Oncol. 2020 Dec 1;4:32. doi: 10.1038/s41698-020-00135-2 (PMC7708412; doi:10.1038/s41698-020-00135-2)
Supplement: Supplementary file 2 — Reporting Summary Checklist [file 41698_2020_135_MOESM2_ESM.pdf]

nature research

Corresponding author(s): DBPR NPJPRECISIONONCOLOGY-00534-T

Last updated by author(s): Jul 24, 2020

Reporting Summary

Nature Research wishes to improve the reproducibility of the work that we publish. This form provides structure for consistency and transparency in reporting. For further information on Nature Research policies, see our Editorial Policies and the Editorial Policy Checklist.

Statistics

For all statistical analyses, confirm that the following items are present in the figure legend, table legend, main text, or Methods section.

n/a	Confirmed	
☒	☐	The exact sample size (*n*) for each experimental group/condition, given as a discrete number and unit of measurement
☒	☐	A statement on whether measurements were taken from distinct samples or whether the same sample was measured repeatedly
☒	☐	The statistical test(s) used AND whether they are one- or two-sided<br>*Only common tests should be described solely by name; describe more complex techniques in the Methods section.*
☒	☐	A description of all covariates tested
☒	☐	A description of any assumptions or corrections, such as tests of normality and adjustment for multiple comparisons
☒	☐	A full description of the statistical parameters including central tendency (e.g. means) or other basic estimates (e.g. regression coefficient) AND variation (e.g. standard deviation) or associated estimates of uncertainty (e.g. confidence intervals)
☒	☐	For null hypothesis testing, the test statistic (e.g. *F*, *t*, *r*) with confidence intervals, effect sizes, degrees of freedom and *P* value noted<br>*Give P values as exact values whenever suitable.*
☒	☐	For Bayesian analysis, information on the choice of priors and Markov chain Monte Carlo settings
☒	☐	For hierarchical and complex designs, identification of the appropriate level for tests and full reporting of outcomes
☒	☐	Estimates of effect sizes (e.g. Cohen's *d*, Pearson's *r*), indicating how they were calculated

Our web collection on statistics for biologists contains articles on many of the points above.

Software and code

Policy information about availability of computer code

Data collection	no software was used.
Data analysis	no software was used.

For manuscripts utilizing custom algorithms or software that are central to the research but not yet described in published literature, software must be made available to editors and reviewers. We strongly encourage code deposition in a community repository (e.g. GitHub). See the Nature Research guidelines for submitting code & software for further information.

Data

Policy information about availability of data

All manuscripts must include a data availability statement. This statement should provide the following information, where applicable:

- Accession codes, unique identifiers, or web links for publicly available datasets
- A list of figures that have associated raw data
- A description of any restrictions on data availability

The slide images of each post-treatment resected specimen in this study are available from the corresponding authors (J.Y. and S.G.) upon reasonable request and through collaborative investigations.

Field-specific reporting

Please select the one below that is the best fit for your research. If you are not sure, read the appropriate sections before making your selection.

☒ Life sciences ☐ Behavioural & social sciences ☐ Ecological, evolutionary & environmental sciences

For a reference copy of the document with all sections, see nature.com/documents/nr-reporting-summary-flat.pdf

Life sciences study design

All studies must disclose on these points even when the disclosure is negative.

Sample size	This is a retrospective study. A total of 31 samples were included. No sample-size calculation was performed.
Data exclusions	No data were excluded from the data analysis.
Replication	This is a retrospective study, and no statistical analysis was used. Replication was not relevant to our study.
Randomization	This is a retrospective study, and no statistical analysis was used. Randomization was not relevant to our study.
Blinding	This is a retrospective study, and no statistical analysis was used. Blinding was not relevant to our study.

Reporting for specific materials, systems and methods

We require information from authors about some types of materials, experimental systems and methods used in many studies. Here, indicate whether each material, system or method listed is relevant to your study. If you are not sure if a list item applies to your research, read the appropriate section before selecting a response.

Materials & experimental systems

n/a	Involved in the study	
☐	☒	Antibodies
☒	☐	Eukaryotic cell lines
☒	☐	Palaeontology and archaeology
☒	☐	Animals and other organisms
☒	☐	Human research participants
☐	☒	Clinical data
☒	☐	Dual use research of concern

Methods

n/a	Involved in the study	
☒	☐	ChIP-seq
☒	☐	Flow cytometry
☒	☐	MRI-based neuroimaging

Antibodies

Antibodies used	(CD20[Clone L26, Ready-to-use, Kit-0001, Maxim Biotechnologies, Fuzhou, China], CD3[Clone SP7, Ready-to-use, Kit-0003, Maxim Biotechnologies, Fuzhou, China], CD4[Clone UMAB64, Ready-to-use, ZM-0418, Zhongshan Golden Bridge Biotechnologies, Beijing, China], CD8[Clone SP16, Ready-to-use, ZA-0508, Zhongshan Golden Bridge Biotechnologies, Beijing, China], CD163[Clone 10D6, Ready-to-use, ZM-0428, Zhongshan Golden Bridge Biotechnologies, Beijing, China], CK[Clone AE1/AE3, Ready-to-use, Kit-0009, Maxim Biotechnologies, Fuzhou, China], PD-L1[Clone 22C3 pharmDx, Ready-to-use, SK006, DAKO])
Validation	Commercial NMPA approved antibodies.

Clinical data

Policy information about clinical studies

All manuscripts should comply with the ICMJE guidelines for publication of clinical research and a completed CONSORT checklist must be included with all submissions.

Clinical trial registration	ChiCTR-OIC-17013726
Study protocol	www.chictr.org.cn
Data collection	The information of data collection is included in the paper published in the Journal of Thoracic Oncology, DOI: 10.1016/j.jtho.2020.01.017
Outcomes	The primary trial outcomes were published separately in Journal of Thoracic Oncology, DOI: 10.1016/j.jtho.2020.01.017
